# The Impact of Fluid Therapy on Glycemic Variation in Non-diabetic Patients Undergoing Laparoscopy

**DOI:** 10.7759/cureus.49240

**Published:** 2023-11-22

**Authors:** João Barbosa, Maria Valentim, Mariana Almeida, Lúcia Vasconcelos

**Affiliations:** 1 Anesthesiology Department, Hospital de Braga, Braga, PRT

**Keywords:** glycemic variation, elective surgery, laparoscopic surgery, fluid therapy, hyperglycemia

## Abstract

Background

Hyperglycemia is a risk factor for perioperative morbidity and mortality. A surgical procedure triggers a physiological stress response, which culminates in insulin resistance by activating the sympathetic autonomic system. The impact of fluid management in the perioperative period on the glycemic variation of patients has not been thoroughly investigated.

Methods

This study, which included 42 non-diabetic patients undergoing laparoscopic surgeries, was an observational, prospective cohort study. The sample was split into two groups according to the type of fluid used intraoperatively: polyelectrolyte and 5% glucose polyelectrolyte.

Results

No significant differences were found between the groups in demographic and baseline data, including age, BMI, and American Society of Anesthesiologists (ASA) physical status. There were no differences in glycemic variation between the two groups. Blood glucose varied over time with statistical significance in the perioperative period but with no difference between the two groups.

Conclusion

Using 5% glucose polyelectrolyte in laparoscopic surgery for non-diabetic patients with ASA physical status 3 or lower did not significantly affect glycemic variation compared to polyelectrolyte. These results suggest the possibility of optimizing resources and minimizing waste without compromising patient homeostasis in perioperative care.

## Introduction

Hyperglycemia is a well-known risk factor for perioperative morbidity and mortality. Surgical procedures, from a physiological perspective, disrupt the patient's homeostasis and induce insulin resistance and a transient catabolic state, even in non-diabetic patients [1,2]. Hyperglycemia resulting from insulin resistance leads to impaired vasodilation and nitric oxide regeneration by the endothelium, increased serum cytokine levels, and impaired neutrophil chemotaxis and phagocytosis, resulting in increased inflammation, susceptibility to infection, and multiorgan dysfunction [3].

The degree of insulin resistance depends on the tissue trauma, with laparotomy causing a more vigorous inflammatory response compared to less invasive techniques such as laparoscopy [4]. One of the well-studied morbidities is the development of surgical site infections, and glycemic control is one of the targets of several perioperative protocols aiming to reduce the risk of healthcare-associated infections, such as the "STOP infection" protocol [5]. The Portuguese General Directorate of Health recommends maintaining blood glucose levels below 180 mg/dL during surgery and in the 24-hour postoperative period to prevent surgical site infections [6]. The estimated physiological glucose requirements to prevent catabolism are 150 g/day in non-diabetic adults, equivalent to 6 g/h. During the perioperative period, due to prolonged fasting and the impact of surgical stress, it is appropriate to administer 5 to 10 g/h of glucose. Intraoperative fluid therapy is a controversial and poorly standardized topic, with limited studies on its impact on glycemic variability and patient outcomes [7].

This study aimed to compare the use of polyelectrolyte with 5% glucose polyelectrolyte, both commonly used in clinical practice without clear scientific evidence of their advantages and their impact on intraoperative glycemic variability in patients undergoing laparoscopic cholecystectomy and colectomy.

While the sympathetic nervous system’s response to surgical stimuli exacerbates insulin resistance, the continuous delivery of external glucose during surgery may mitigate these catabolic effects, resulting in less pronounced glycemic fluctuations. It is important to note that not only can the absolute maximum intraoperative blood glucose level have consequences for patient morbidity and mortality, but also glycemic variability itself can lead to increased oxidative stress compared to stable blood glucose levels [8].

The primary endpoint of the study was the evaluation of capillary blood glucose levels at baseline (prior to surgical incision) and hourly intraoperatively, with a final postoperative assessment (1 hour after the end of surgery). The obtained values were compared between the groups receiving polyelectrolyte versus 5% glucose polyelectrolyte.

## Materials and methods

Following approval by the Ethics Committee of Hospital de Braga (CEHB), numbered approval nº97_2021, this observational analytic cohort study was conducted in November 2021 at Braga’s Hospital, Braga, Portugal. Patients scheduled for elective laparoscopic cholecystectomy or colectomy were selected and divided into two groups according to the fluid therapy administered during surgery: one group receiving polyelectrolyte and the other group receiving 5% glucose ​​polyelectrolyte. All patients received informed consent and had it explained to them.

The inclusion criteria for this study were as follows: age over 18 years; surgical indications for elective laparoscopic cholecystectomy or colectomy; non-diabetic patients; administration of dexamethasone (4 mg) for antiemetic prophylaxis to standardize frequent drugs with hyperglycemic effects in the sample; and the American Society of Anesthesiologists physical status (ASA PS) 1 to 3 [9].

The following patients were excluded: patients diagnosed with diabetes mellitus (as it has intrinsic pathophysiological characteristics that affect glycemic hormonal regulation); patients with a body mass index (BMI) > 35; patients with an ASA physical status > 3; any laparoscopic surgery performed in an emergency setting, laparoscopic surgeries converted to laparotomies, and patients who did not receive dexamethasone as intraoperative antiemetic prophylaxis. Patients with blood glucose levels > 200 mg/dL during surgery requiring insulin therapy were also excluded.

All patients received fluid therapy according to their respective groups with a dial flow infusion kit at a rate of 140 mL/h, based on glucose administration calculations of 7 g/h for the 5% glucose polyelectrolyte group. Capillary blood glucose levels were measured in the participants at baseline (prior to surgical incision) and hourly intraoperatively, with a final postoperative assessment (1 hour after the end of surgery) in the Post-Anesthesia Care Unit (PACU).

The intraoperative glycemic variability of each patient was evaluated and classified according to the type of fluid used. The primary statistical endpoint was to evaluate whether there were statistically significant differences in the mean glycemic variability between the two groups. After checking the normal distribution of the variables in both groups, a student's t-test was conducted to assess if age, BMI, and ASA PS had statistically significant differences between groups. To assess the differences in the progression over time of the two study groups, a repeated measures within-between interaction analysis of variance (ANOVA) analysis was used. The main effects and interactions were evaluated. The null hypothesis was rejected for a p-value less than 0.05, and post-hoc tests were performed to determine the direction of the differences found. Considering the repeated measures within-between interaction ANOVA test with two groups and three measurements, a moderate effect size of 0.20(f), a significance level of 0.05, and a statistical power of 0.80, a sample size of 42 participants (21 in each group) was required. Allocation among groups was done in order of surgical listing of patients, which was considered random, alternating a new patient to each group until we met the necessary sample according to the defined statistical power. The statistical analysis was performed using GraphPad Prism version 9.0 software.

## Results

Three patients were excluded from the study due to intraoperative blood glucose levels exceeding 200 mg/dL. Of these three patients, one was receiving 5% glucose polyelectrolyte (P+G5%) and two were receiving polyelectrolyte (P).

The characteristics of the patients between groups were analyzed to evaluate any differences that could potentially bias the final outcome.

The mean age, BMI, and ASA physical status did not show statistically significant differences between the two groups (Table 1), indicating that the groups had a homogeneous distribution of these characteristics.

**Table 1:** Characteristics of both groups and application of the student's t-test to evaluate differences between them ASA PS - American Society of Anaesthesiologists’ Physical Status; BMI - Body Mass Index; P - Polyelectrolyte; P+G5% - Polyelectrolyte + Glucose 5%; The data have been represented as Mean±SD; p-value is considered significant if p<0.05

	Group P+G5% (n=21)	Group P (n=21)	Student's t-test (p-value)
BMI (kg/m²)	26.66 ± 4.27	27.73 ± 4.79	0.45
Age (years)	59.05 ± 17.15	62.33 ± 13.47	0.46
ASA PS	2.10 ± 0.62	2.24 ± 0.62	0.49

The distribution of the types of surgeries between the two groups is described in Table 2.

**Table 2:** Distribution of types of surgeries in both groups P - Polyelectrolyte; P+G5% - Polyelectrolyte + Glucose 5%; The data have been represented as N and % within group

Group P+G5%	
Colectomy	5 (23.8%)
Cholecystectomy	10 (47.6%)
Anterior rectal resection	6 (28.6%)
Group P	
Colectomy	10 (47.6%)
Cholecystectomy	8 (38.1%)
Anterior rectal resection	3 (14.3%)

All 42 surgeries lasted for more than one hour, which allowed for a baseline value, a value at one hour of surgery, and a value after one hour of surgery in the entire sample. However, only 50% (n=21) of the surgeries lasted for more than two hours, with 10 patients in the polyelectrolyte + G5% group and 11 in the polyelectrolyte group.

**Figure 1:**
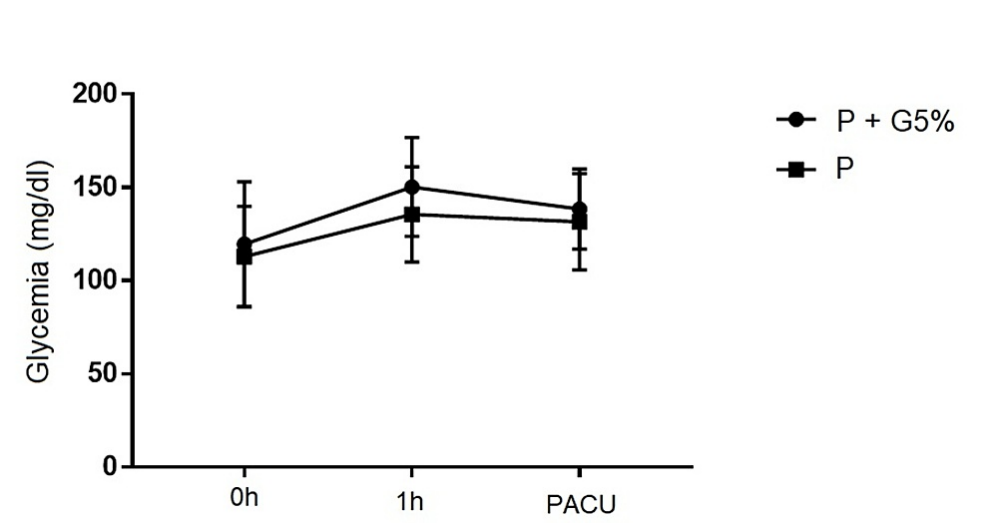
Variation of glucose levels in both groups (P and P+G5%) PACU - Post-Anesthesia Care Unit; P - Polyelectrolyte; P+G5% - Polyelectrolyte + Glucose 5% The data have been represented as mean±SD. The p-value is considered significant if p<0.05.

The test for differences in means and variations of glucose levels in the 42 surgeries for baseline, 1-hour, and 1-hour postoperative values did not show statistically significant differences between the two types of fluid therapy (p>0.05). Overall, glucose levels varied over time with statistical significance but without a difference between the two groups (Figure 1). ANOVA repeated measures: Group/Time interaction F(2,80)=0.58, p=0.56; Time F(2,80)=21.25, p < 0.0001; P/P+G5% F(1,40)=1.96; p=0.17.

**Figure 2:**
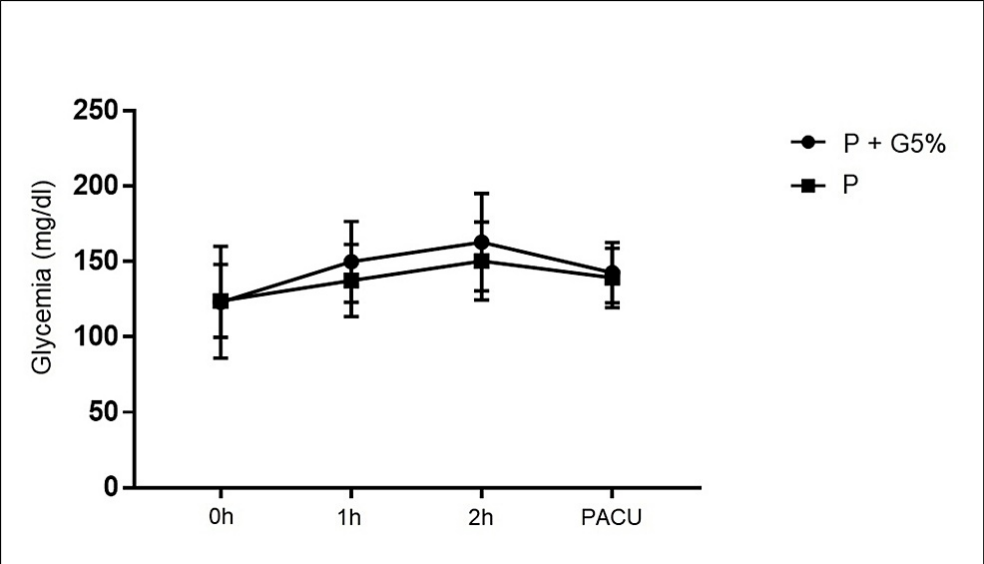
Variation of glucose levels in the 21 surgeries lasting more than 2 hours in both groups (P and P+G5%) PACU - Post-Anesthesia Care Unit; P - Polyelectrolyte; P+G5% - Polyelectrolyte + Glucose 5% The data have been represented as Mean±SD. The p-value is considered significant if p<0.05.

The same analysis was conducted for the 21 surgeries lasting more than 2 hours in order to assess whether the additional cumulative effect of one more hour of surgical stress would lead to significant differences between these two types of fluid therapy. As shown in Figure 2, there were no statistically significant differences, even in the longer surgeries, between the two groups. Overall, glucose levels varied statistically significantly over the course of the surgery, and this variation was not dependent on the type of fluid therapy administered.

ANOVA repeated measures: Group/Time interaction F(3,57)=0.59, p=0.62; Time F(3,57)=9.86, p < 0.0001; P/P+G5% F(1,19)=0.59; p=0.45.

## Discussion

Perioperative fluid therapy management is a controversial theme because of its importance for optimal outcomes after surgery. The pursuit of the right fluid and its precise quantity in the intraoperative setting is an ongoing debate. The end goal is to avoid hypovolemia and hypervolemia due to its postoperative complications [10].

In the pediatric population, it is accepted that in order to strike a balance between the risks of hypoglycemia and hyperglycemia, isotonic fluids with low glucose concentration (1-2.5%) should be used [11]. However, in the adult population who is submitted to elective surgery and commonly exposed to longer fasts than needed [12,13], this rationale seems to be underestimated. Insulin resistance and a temporary catabolic state, even in individuals without diabetes, is a well-known consequence of the stress response caused by surgical procedures [1,2]. Since muscle wasting during a catabolic state may lead to postoperative complications [14], the use of a glucose infusion perioperatively may have its benefits since its action in suppressing muscle breakdown has been established in previous studies [15-17].

This study demonstrated that, for a non-diabetic population with ASA physical status equal to or inferior to 3, the use of 5% glucose polyelectrolyte did not significantly alter glycemic variation in laparoscopic surgery when compared to simple crystalloids.

The results of previous studies are conflicting. In a study involving patients undergoing neurosurgical procedures, the administration of fluids containing dextrose maintained blood glucose levels within acceptable limits while avoiding the risk of hypoglycemia in some patients [18].

On the other hand, there are studies that show statistically significant differences in intraoperative blood glucose levels between groups that received fluids with and without dextrose, increasing the need for insulin administration in the dextrose groups [19,20].

At our hospital, it is common for patients admitted for surgery to be prescribed glucose-containing fluid therapy at a slow maintenance rate that is not quantified. Upon arrival in the operating room, it is very frequent for the anesthesiologist to decide to switch to a crystalloid solution without glucose, with the glucose solution being discarded and often underutilized due to the slow rate at which it is administered during the hospital stay, leading to unnecessary expenses and waste of these products.

Our study results may lead to a change in standardized practices in our daily hospital routine, demonstrating that for a considerable population undergoing elective laparoscopic surgery, it is possible to optimize resources and avoid waste without compromising patient homeostasis in the perioperative period by maintaining the 5% glucose polyelectrolyte in patients with these characteristics.

In the perioperative period, euvolemia should be maintained to achieve adequate tissue perfusion. Both fluid deficit and excess are associated with perioperative morbidity such as acute kidney injury, pulmonary edema, suture dehiscence, and increased risk of postoperative nausea and vomiting [10]. Liberal fluid therapy or fixed-volume replacement strategies have been abandoned due to the likelihood of fluid overload [21]. Therefore, in the evaluated context where patients with controlled comorbidities undergo elective laparoscopic surgeries with expected low blood loss, a more restrictive fluid therapy aimed at maintaining hemodynamic stability, as used in this study, allows for the use of glucose-containing solutions without altering blood glucose levels during the procedure. It should be noted that in the event of an anesthetic-surgical complication requiring a fluid challenge or significant blood loss replacement, the use of a glucose-containing crystalloid would lead to undesirable hyperglycemia.

Regarding the pathophysiology of surgical stress, the obtained results seem to raise new questions. Alpha-adrenergic stimulation by the sympathetic nervous system is responsible for the insulin resistance associated with the surgical stimulus. It would be logical to deduce that in this transitory period of insulin resistance, maintaining an external source of glucose would increase the patient's blood glucose levels compared to the crystalloid without glucose group. However, as this was not observed in this study, the hypothesis arises that the provision of external glucose may mitigate this acquired perioperative insulin resistance state, contributing to a less pronounced manifestation of surgical stress.

To the best of our knowledge, this is the first prospective study comparing polyelectrolytes with 5% glucose polyelectrolytes concerning glycemic variation in the intraoperative period of a laparoscopic procedure. This study should be interpreted in the context of its limitations. First of all, this research was conducted at a single institution and had a limited number of participants. In a study involving multiple centers, a larger number of participants could enhance the statistical strength of the investigation. Second, the anesthesiologist in the operating room was not blinded to the patient group assignment. Also, this study was not randomized, so it is difficult to have definitive conclusions about our results.

## Conclusions

This prospective study examined the effects of polyelectrolyte or 5% glucose polyelectrolyte perfusions on hyperglycemia fluctuation in the perioperative period. The findings imply that there is no difference in glycemic variation with the administration of polyelectrolyte or 5% glucose polyelectrolyte in non-diabetic patients with ASA physical status ≤ 3 undergoing elective laparoscopic surgery under restrictive fluid treatment. By exchanging crystalloid solutions for optional treatments, waste is decreased and resources can be used more efficiently as a result. To corroborate these findings, additional research in various patient demographics and surgical techniques is required.
